# 842. Regenerative and Antibacterial Properties of Decellularized Pisces Dermis for Treatment of Fournier’s & Perineal Wounds

**DOI:** 10.1093/jbcr/irag033.281

**Published:** 2026-04-06

**Authors:** Alfredo C Cordova, Victoria Young, Kristy Miller, Talia Selembo

**Affiliations:** Sarasota Memorial Hospital, Sarasota, FL; Sarasota Memorial Hospital, Sarasota, FL; Sarasota Memorial Hospital, Sarasota, FL; Sarasota Memorial Hospital, Sarasota, FL

## Abstract

**Introduction:**

Fournier’s gangrene may be a life threatening condition. Furthermore, perineal wounds are challenging to manage due to their location, irregular surface, drainage control, and high bacterial colonization. Most skin-substitutes are highly sensitive to bacterial colonization and infection. Decellularized and lyophilized fish dermis (DLFD) have been shown in-vitro to possess effects decreasing bacterial migration and proliferation acting as a bacterial barrier. DLFD may serve with bacterial protection and enhance optimal wound regeneration in preparation for grafting.

**Methods:**

Eight patients with numerous comorbidities sustaining full-thickness skin defects and complicated wounds with at least heavy bacterial colonization were included. These patients had sustained necrotizing soft tissue infections to the perineal and perianal regions. They underwent excisional debridement and local wound care. Application of DLFD and negative pressure wound therapy was then performed. Subsequently, they underwent resurfacing with a split-thickness skin graft (STSG).

**Results:**

Despite the location, challenges on dressings application, and bacterial colonized environment complete Xenograft incorporation and wound enhancement for grafting was noted within 10 to 14-days. Graft integration and optimal granulation tissue was evidenced in >95% surface area as early as 7-days after product application. No graft loss occurred. Subsequent, STSG revealed >95% graft-take and epithelization within 2-3 weeks.

**Conclusions:**

DLFD provide excellent wound coverage of perineal colonized wounds, act as bacterial barrier, and enhances formation of optimal wound bed for skin-grafting. Even though these properties have been observed, we do not advocate using any skin substitute on an infected field. Adequate wound bed preparation is paramount for the success of our patients.

**Applicability of Research to Practice:**

Acellular Fish dermis provide excellent wound coverage of perineal wounds, may have antibacterial properties, and accelerates the formation of optimal wound bed for skin-grafting.

**Funding for the study:**

N/A.

